# PIM-1 modulates cellular senescence and links IL-6 signaling to heterochromatin formation

**DOI:** 10.1111/acel.12249

**Published:** 2014-07-18

**Authors:** Bo Jin, Yu Wang, Chen Lin Wu, Kai Yu Liu, Hao Chen, Ze Bin Mao

**Affiliations:** 1 Department of Biochemistry and Molecular Biology, Health Science Center, Peking University38 Xueyuan Road, Beijing, 100191, China; 2 Department of Microbiology, School of Medicine, New York University550 First Avenue, New York, NY, 10016, USA

**Keywords:** HP1γ, IL-6, PIM-1, senescence-associated heterochromatin foci, senescence, STAT3

## Abstract

Cellular senescence is a stable state of proliferative arrest that provides a barrier against malignant transformation and contributes to the antitumor activity of certain chemotherapies. Unexpectedly, we found that the expression of proto-oncogene PIM-1, which can promote tumorigenesis, is induced at transcriptional level during senescence. Inhibition of PIM-1 alleviated both replicative and oncogene-induced senescence. Conversely, ectopic expression of PIM-1 resulted in premature senescence. We also revealed that PIM-1 interacts with and phosphorylates heterochromatin protein 1γ (HP1γ) on Ser93. This PIM-1-mediated HP1γ phosphorylation enhanced HP1γ’s capacity to bind to H3K9me3, resulting in heterochromatin formation and suppression of proliferative genes, such as CCNA2 and PCNA. Analysis of the mechanism underlying the up-regulation of PIM-1 expression during senescence demonstrated that IL-6, a critical regulator of cellular senescence, is responsible for PIM-1 induction. Our study demonstrated that PIM-1 is a key component of the senescence machinery that contributes to heterochromatin formation. More importantly, we demonstrated that PIM-1 is also a direct target of IL-6/STAT3 signaling and mediates cytokine-induced cellular senescence.

## Introduction

Senescence was initially described as a stable cell proliferation arrest resulting from the progression of primary human fibroblasts through a finite number of populations doubling *in vitro* (Hayflick, 1965). However, the definition of senescence has since been broadened, stating that DNA damage and cellular stress, caused by expression of certain oncogenes, unscheduled DNA replication, oxidative stress, or dysfunctional telomeres, can both induce cellular senescence (Nishiyama *et al*., 1999; Collado *et al*., 2007). Senescence is an important tumor suppression mechanism that restrains the proliferation of cells harboring activated oncogenes (Braig *et al*., 2005; Chen *et al*., 2005; Collado *et al*., 2005). Also, by depleting renewable stem cell populations, senescence is thought to contribute to tissue aging of many multicellular adult animals (Janzen *et al*., 2006; Krishnamurthy *et al*., 2006; Molofsky *et al*., 2006).

Previous studies have proposed that the irreversibility of the senescence arrest is tightly associated with particular chromatin architecture in senescent cells, called senescence-associated heterochromatin foci (SAHF) (Narita *et al*., 2003; Adams, 2007). Although not all cells exhibit such dramatic heterochromatinization, some degree of heterochromatinization appears to be a general feature of senescence in various cell types from several species (Braig *et al*., 2005; Denoyelle *et al*., 2006; Herbig *et al*., 2006). SAHF associates with transcriptional silence and coincides with the recruitment of heterochromatin proteins, such as methylated lysine nine of histone H3 (H3K9Me), heterochromatin protein 1 (HP1) and histone variant macroH2A (Narita *et al*., 2003; Zhang *et al*., 2005), and some other proteins that are less typically linked to heterochromatin, such as high-mobility group A (HMGA) proteins (Funayama *et al*., 2006; Narita *et al*., 2006). Formation of SAHF in human is driven by a complex of histone chaperones, including histone repressor A (HIRA) and antisilencing function 1a (ASF1a) that play an evolutionarily conserved role in heterochromatin formation through plants, yeast, and other mammals (Zhang *et al*., 2005). Proliferation-promoting genes, such as E2F-targeting gene CLNA, are recruited into SAHF, resulting in an irreversibly silence of gene expression (Narita *et al*., 2006). Consequently, heterochromatinization appears to be important for senescence-associated tumor suppression (Narita *et al*., 2006).

PIM-1 is a proto-oncogene, encoding a serine/threonine protein kinase that regulates cell proliferation and growth. Overexpression of PIM-1 leads to tumor formation in mice, while complete PIM-1 knockout has no observable phenotype. PIM-1 expression in normal tissues is nearly undetectable. However, in hematopoietic malignancies and in many solid tumors, increased PIM-1 expression has been shown to correlate with the stage of disease (Nawijn *et al*., 2011). Mechanism analysis showed that PIM-1 contributes to cancer development by phosphorylating multiple target substrates that are tightly related to cells proliferation and anti-apoptosis (Bachmann & Moroy, 2005). Oncogenic characteristic of PIM-1 makes it an attractive target for cancer therapy (Bachmann & Moroy, 2005; Magnuson *et al*., 2010; Nawijn *et al*., 2011).

In this study, we demonstrated that Pim1 acts as a positive regulator of cellular senescence, which implies that Pim1 likely has characteristics of tumor suppressor. Significantly, we demonstrated that PIM-1 is a direct target of IL-6/STAT3 signaling and mediates cytokine-induced cellular senescence.

## Results

### PIM-1 expression is up-regulated in senescent cells

**Figure 1:**
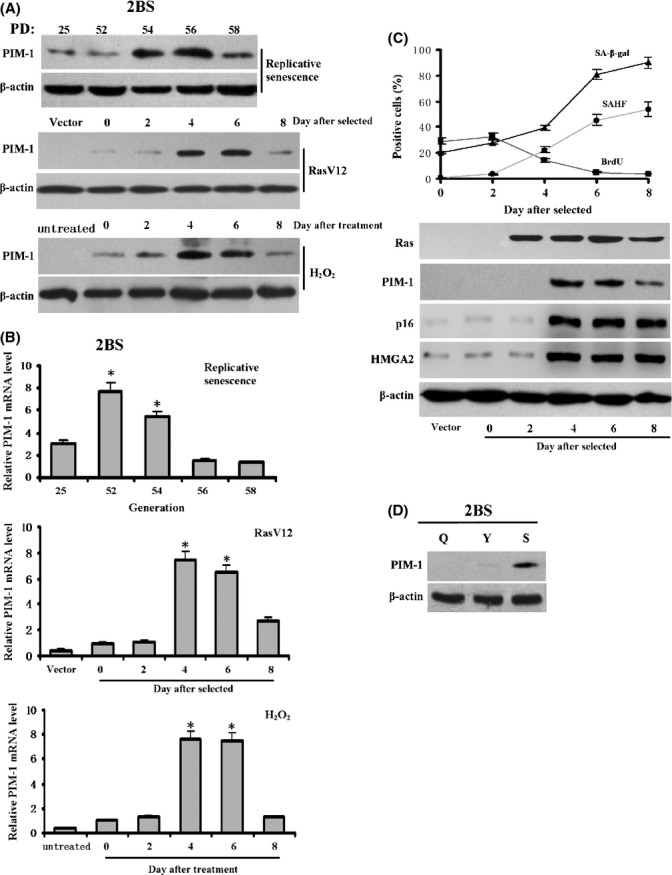
PIM-1 expression is up-regulated in senescent 2BS Cells. (A, B) Western blot and real-time PCR analysis of PIM-1 expression in replicative senescent, Ras-induced and H_2_O_2_-induced premature senescent 2BS cells. (C) Ras-induced senescent 2BS cells were assessed for SA-β-gal, BrdU incorporation, SAHF formation, and expression of the indicated proteins at the various time points after RasV12 selected. Each value of SA-β-gal, BrdU incorporation, and SAHF formation represents the mean ± SD. p16 and HMGA2 serve as markers of senescence. (D) Western blot analysis of PIM-1 expression in 2BS cells that are young (Y), quiescent (Q) by serum-deprivation, and senescent (S) by Ras induction. ß-actin was used as loading control. Data are expressed as the mean ± SD of three independent experiments unless notice otherwise. * *P* < 0.05 (*t*-test).

To determine whether PIM-1 is involved in senescence, we started with the measurement of PIM-1 expression during senescence in 2BS human diploid fibroblasts (HDF). Real-time PCR and western blots showed that PIM-1 expression was swiftly induced at population doublings (PD) 54 during replicative senescence and then gradually decreased after PD58 (Fig. 1A,B). A similar kinetic change in PIM-1 expression was observed in RasV12 and H_2_O_2_-induced premature senescence (Fig. 1A,B). By comparing PIM-1 expression with senescence markers in RasV12-induced senescence, we found that the induction of PIM-1 expression occurred when cells just entered senescence (Fig. 1C). There was no increase in PIM-1 expression in quiescent cells (Fig. 1D), suggesting that the increase in PIM-1 expression is associated with senescence rather than with cell cycle arrest. Similar results were obtained in another HDF cell, WI-38 (Fig. S1A–C), suggesting that PIM-1 up-regulation is not unique to 2BS senescence. We did not observe significant change in PIM1 or p16 expression in control group without RasV12 (Fig. S1D).

### PIM-1 depletion delays cellular senescence

**Figure 2:**
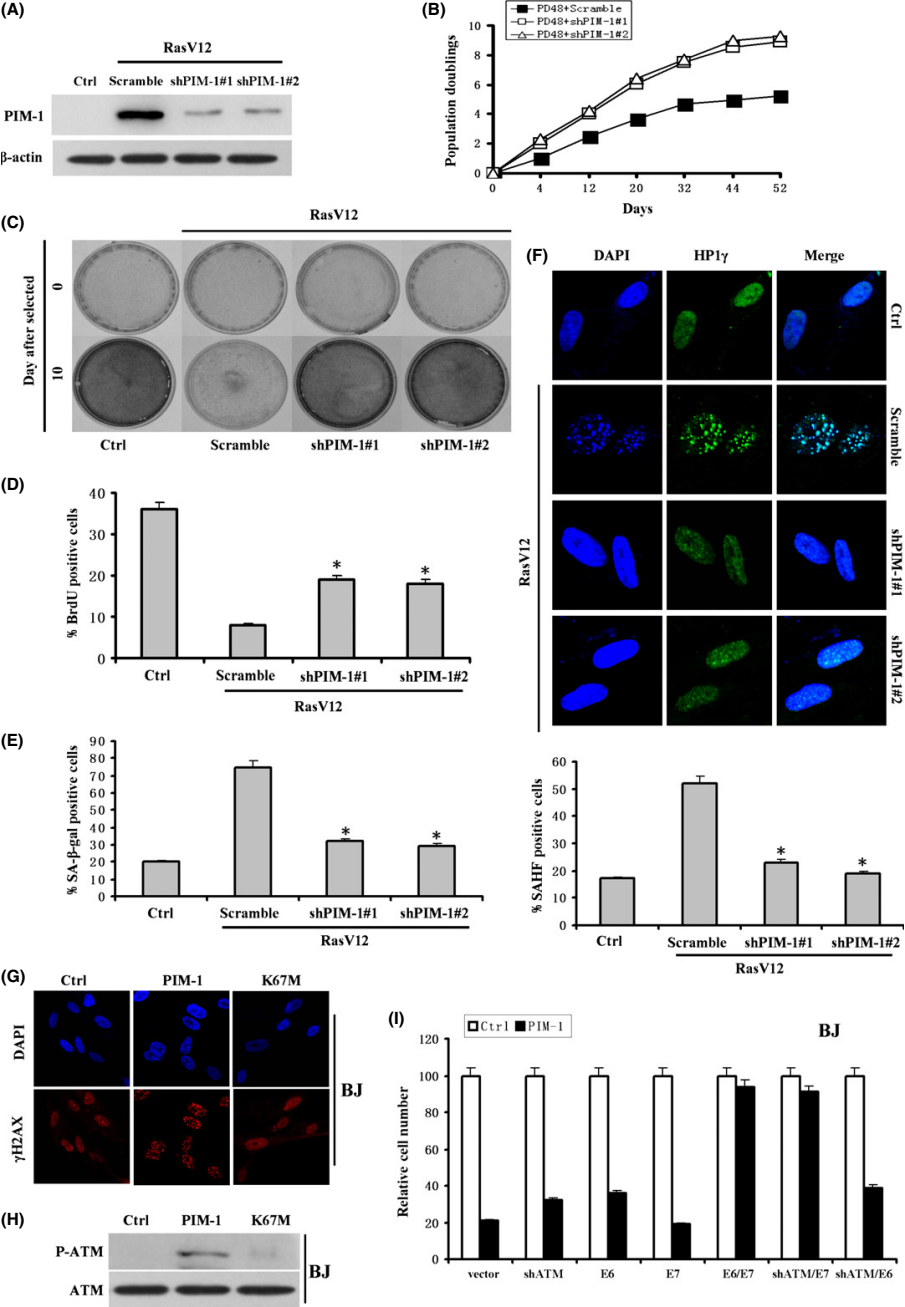
PIM-1 depletion delays cellular senescence. (A) Western blot of PIM-1 expression in 2BS cells expressing PIM-1 shRNA#1 or shRNA#2 at 12 days after exposure to RasV12. (B) 2BS cells at PD 30 were infected with lentivirus expressing PIM-1 shRNA#1 or shRNA#2 or scramble, after selection growth curves were performed. (C) Cells proliferation assay of polyclonal 2BS expressing independent, nonoverlapping shRNA against PIM-1 upon exposure to RasV12. A representative experiment out of three independent experiments is shown. (D) BrdU incorporation of 2BS cells expressing PIM-1 shRNAs upon exposure to RasV12. Cells expressing scramble or PIM-1 shRNAs were infected with RasV12 retroviruses, and labeling was conducted at 5 days after selection. *, *P* < 0.05 (*t*-test). Same cells as (D) were analyzed for SA-β-gal activity (E) and SAHF formation (F). (F), Immunofluorescence was performed for HP1γ (green), and DAPI staining (blue) was used to visualize DNA (upper panel). Percentages of SAHF-positive cells are indicated in lower panel. *, *P* < 0.05 (*t*-test). (G) Indirect immunofluorescence for γH2AX foci in BJ cells. (H) BJ cells expressing the control vector or wtPIM-1 or K67M were analyzed for expression of p-ATM proteins by western blot. (I) BJ cells infected with the indicated vectors were seeded. After 10 days, the plates were fixed and stained with crystal violet. Crystal violet was extracted and quantified.

To investigate whether PIM-1 is causally involved in replicative senescence, we used two independent validated short hairpin (shRNA) targeting PIM-1. Cells at PD30 were transduced with these shRNA-expressing lentiviruses, selected for proviral integration, and plated at equal densities. As shown in Fig. 2A, both shRNAs caused a 75% reduction of PIM-1 expression as judged by western blot. Depletion of PIM-1 with either shRNA delayed senescence in 2BS cells without cell immortalization (Fig. 2B).

Next, we tested whether knockdown of PIM-1 influences RasV12-induced premature senescence. PIM-1 was knocked down by two shRNAs, as described above, while senescence was induced by activated RasV12. A cell proliferation assay showed that PIM-1 depletion delays RasV12-induced cellular senescence (Fig. 2C). Similar results were achieved with two independent shRNAs (shPIM-1#1 and shPIM-1#2) (Fig. 2C). The percentage of cells incorporating BrdU also increased upon knockdown of PIM-1 as measured on day 5 after drug selection (Fig. 2D). Furthermore, PIM-1 knockdown resulted in a reduced percentage of cells with senescence-associated β-galactosidase (SA-β-gal) staining (Fig. 2E) and SAHF formation (Fig. 2F), two well-known markers of cellular senescence. These data indicate that PIM-1 is associated with cells senescence. Similar results were observed following transfection of controls and shPIM-1s into BJ fibroblasts infected with a retrovirus-encoding H-RasV12 (Fig S3B).

To complement the findings with PIM-1shRNAs, we then investigated whether overexpression of PIM-1 would induce premature senescence. Early passage 2BS human diploid fibroblasts (PD20) were infected with pBABE-puro (Ctrl), retrovirus vector expressing wild-type PIM-1 (wtPIM-1), or kinase-dead PIM-1 mutant (K67M). Cells overexpressing wtPIM-1 showed proliferation arrest (Fig. S2A) and reduced BrdU incorporation (Fig. S2B). PIM-1 also induced characteristic features of senescence, such as ß-gal activity and SAHF formation (Fig. S2C,D). Previous study suggested that p53 plays an important role in PIM-1’s effect on the cell cycle (Zemskova *et al*., 2010). PIM-1 has also been shown to mediate neuronal death upstream of Cdc25A and Cdk/Rb/E2F (Zhang *et al*., 2010). Here, we found that PIM-1 overexpression increased p53, p21, p15, and p16 expression combined with decreased Rb phosphorylation levels, indicating an elevated senescence (Fig. S2E). In contrast, cells infected by retrovirus expressing K67M did not display senescent phenotype, suggesting that PIM-1 induction of senescence is dependent on its kinase activity. Similar results were obtained following infection of BJ fibroblast with PIM-1-expressing retrovirus (Fig. S2E).

Because DNA damage signaling pathway acts as a critical mediator in oncogenes-induced senescence and p53, an important regulator of senescence pathway, increases in premature senescence, we speculated that this pathway also contributes to oncogene PIM-1-induced senescence. Indeed, BJ fibroblasts that senesced in response to PIM-1-accumulated DNA damage foci, which judged by γH2AX immunofluorescence staining and phosphorylated active form of ATM, indicating that aberrant oncogene activation induces a DNA damage signaling response (Fig. 2G,H). Depletion of ATM with an already validated shRNA (shATM) led to reduced p53 accumulation compared to control hairpin, confirming that p53 induction results from activation of DNA damage signaling (Fig S3A). To further explore the role of DNA repair pathway in PIM-1-mediated cell cycle arrest, we analyzed the effects of PIM-1 in BJ fibroblasts in which ATM, p53, or p-Rb functions were disrupted using shRNA, HPV E6, and E7 proteins, respectively (Fig. 2I). The shATM, E6, or E7 alone did not bypass the senescent response to PIM-1 in BJ fibroblasts (Fig. 2I). However, co-expressing both E6 and E7 did circumvent PIM-1-induced senescence (Fig. 2I). Similarly, combining shATM with E7 but not with E6 also blocked PIM-1-induced senescence (Fig. 2I). These data clearly indicated the importance of DNA damage signaling pathway as well as the Rb pathway in establishing the PIM-1-induced arrest in human cells.

### PIM-1 knockdown diminishes the DNA damage response of cells to RasV12

Because PIM-1 is a downstream target of active RasV12, we next investigated whether PIM-1 modulates RasV12-induced DNA damage response. To this end, we exposed BJ cells infected with a control or shPIM-1 to RasV12. PIM-1 knockdown led to decreased levels of γH2AX, phosphorylated ATM, and p53 in RasV12-induced senescent cells (Fig. S4A). In contrast to PIM-1-induced senescence, RasV12-induced senescence is more complex and cannot be entirely rescued with E6 and E7. Co-expression of RasV12 with shPIM-1 and E7 or shPIM-1 and E6 did not prevent cells senescence. By comparing the effect of E6, E7, and shPIM-1 co-expressing, separately, on RasV12-induced senescence, we found that shPIM-1/E6 and shPIM-1/E7 were inferior to E6/E7 in delaying RasV12-induced senescence (Fig. S4B), suggesting that PIM-1 only partly contributes to Ras-induced DNA damage.

### PIM-1 interacts with and phosphorylates HP1γ in senescent cells

**Figure 3:**
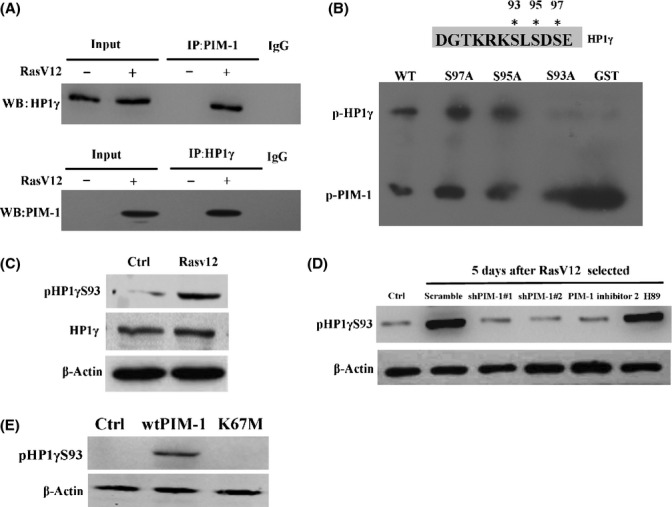
PIM-1 interacts with and phosphorylates HP1γ in senescent cells. (A) Immunoprecipitation analysis of PIM-1 interaction with HP1γ in senescent cells. Cell nuclear extracts from young and Ras-induced senescent 2BS cells were immunoprecipitated with antibodies against the indicated proteins, followed by immunoblotted using antibodies against the indicated proteins (monoclonal mouse anti-human HP1γ antibodies and polyclonal rabbit anti-human PIM-1 antibodies). Input = 12% of the protein. (B) *In vitro* kinase assay of HP1γ phosphorylation by PIM-1. GST by itself and GST fusion proteins of wild-type HP1γ (WT) or mutant HP1γ (S93A, S95A, and S97A) were expressed in *E. coli*, purified, and incubated in the presence of [γ-^32^P] ATP and recombinant PIM-1. (C) Western blots of HP1γ phosphorylation in ras-induced senescent cells with antibody against phospho-HP1γ at S93 (pHP1γS93). Total HP1γ and β-ACTIN served as controls. (D) pHP1γS93 levels were detected by western blot in 2BS cells expressing scramble control, PIM-1 shRNA, or in presence of PIM-1 inhibitor 2 (91 nm) or H89 (5 μm) upon exposure to RasV12. (E) 2BS cells expressing the control vector, wtPIM-1, or K67M were analyzed for expression of the pHP1γS93 levels by western blot.

It has been shown that HP1γ, which plays an important role in heterochromatin formation, acts as target protein of PIM-1, and we hypothesized that besides of effecting DNA damage response, PIM-1 also can potentially affect heterochromatin formation by interacting with and phosphorylating HP1γ protein. At first, we tested whether there is a physical interaction between PIM-1 and HP1γ in oncogene-induced senescence (OIS). To this end, we carried out immunoprecipitation (IP) followed by western blot assay using nuclear extracts of senescent cells. As shown in Fig. 3A, IP with anti-PIM-1 antibody successfully pulled down HP1γ, while reciprocal experiment using anti-HP1γ antibody validated the interaction between PIM-1 and HP1γ in senescent cells.

Because the PIM-1-mediated phosphorylation site in HP1γ has never been identified, we examined which amino acid residue in HP1γ is phosphorylated by PIM-1. In human HP1γ, a putative PIM-1 consensus sequence is located in the amino acid sequence from 90 to 98, ^90^KRKSLSDSE^98^. To identify the residue that is phosphorylated by PIM-1, each of the serine residues in this sequence was mutated to alanine to generate HP1γ (S93A), HP1γ (S95A), and HP1γ (S97A). GST-tagged wild-type HP1γ and mutant HP1γ were expressed and purified from *E. coli* and were incubated with recombinant PIM-1 (rPIM-1) and [γ-^32^P] ATP *in vitro*. Phosphorylation of HP1γ was evidently blocked by the S93A mutation (Fig. 3B). In contrast, the S95A and S97A mutations had no effect on HP1γ phosphorylation (Fig. 3B). These findings suggest that PIM-1 phosphorylates HP1γ on serine 93, which is consistent with the observation that HP1γ is phosphorylated on serine 93 in senescent cells (Zhang *et al*., 2007). For *in vivo* analysis, an affinity-purified antibody specific to the phosphor-peptide KRKpSLSDSE was generated. This antibody specifically recognized the recombinant HP1γ isoform only after phosphorylation in a PIM-1-dependent manner (Fig. S5). Figure 3(C) showed that the pHP1γS93 level clearly increased in RasV12-induced senescent cells compared with the control cells. In addition, ectopically expressing PIM-1-induced cell senescence accompanied by an elevated level of pHP1γS93 (Fig. 3E). To further demonstrate that HP1γ phosphorylation stems from PIM-1 activation, PIM-1 was knocked down by two shRNAs. As shown in Fig. 3D, PIM-1 knockdown decreased the pHP1γS93 level in RasV12-induced senescent cells. A specific PIM-1 inhibitor resulted in a similar inhibition of HP1γ phosphorylation (Fig. 3D). The PIM-1 phosphorylation site overlaps with a previously reported PKA phosphorylation site in immortal and transformed cells (Lomberk *et al*., 2006). It seems unlikely that phosphorylation of HP1γ on this residue resulted from PKA in senescent cells, as the specific PKA inhibitor, H89, did not change the pHP1γS93 level (Fig. 3D). Furthermore, PKA activity is decreased in senescent cells (Fig. S6). Together, these data indicate that PIM-1 directly phosphorylates HP1γ on Ser93 in senescent cells.

### PIM-1-induced HP1γ phosphorylation on Ser93 promotes heterochromatin formation in OIS

**Figure 4:**
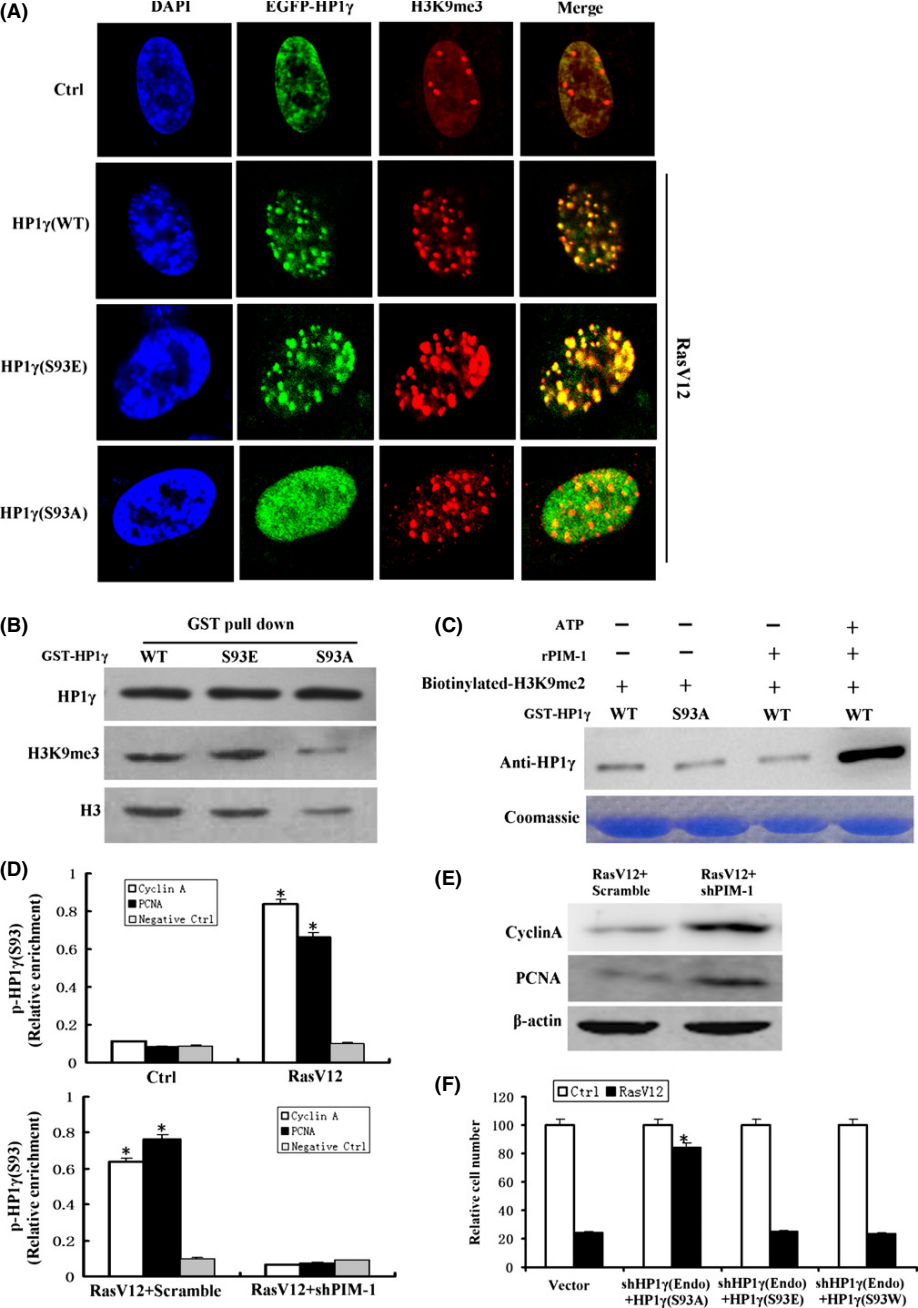
HP1γ phosphorylation by PIM-1 promotes heterochromatin formation in Ras-induced senescent cells. (A) Localization of wild-type (WT) (top panel) or mutant EGFP-HP1γ (S93E, middle panel, or S93A, bottom panel) upon exposure to RasV12. (B) Western blot of proteins pulled down with GST-tagged HP1γ wild-type, S93E or S93A mutant proteins from nuclear extracts of ras-induced senescent cells. Protein pull-down was probed with antibodies against HP1γ (top row), histone H3K9me3 (middle row), or histone H3 (bottom row). (C) *In vitro* analysis of the binding of HP1γ to H3K9m2 peptide in presence of rPIM-1. Coomassie staining shows equivalent loading of GST-HP1γ in each track. (D) ChIP analysis of phosphorylated HP1γ (pHP1γS93) binding to the *CCNA2* and *PCNA* promoter in 2BS cells that expressing vector (Ctrl) or RasV12 (RasV12) or co-expressing shPIM-1 with RasV12 (RasV12 + shPIM-1) or scramble with RasV12 (RasV12 + Scramble). ChIP enrichment was measured using real-time PCR, normalized by input DNA. The *ACTIN* promoter was used as nonresponsive negative control. *P < 0.05. (E) Western blot of cyclin A and PCNA expression in 2BS cells co-expressing scramble with RasV12 or shPIM-1 with RasV12. (F) 2BS cells infected with the indicated vectors were seeded in 10-cm dishes. The plates were fixed 10 day after seeding and stained with crystal violet. Crystal violet was extracted and quantified. *P < 0.05.

We further investigated the impact of PIM-1-induced Ser93 phosphorylation on HP1γ functions. Two mutants were generated by altering Ser93 in HP1γ to Ala or Glu (expected to preclude or mimic phosphorylation, respectively). EGFP-tagged HP1γ (wild-type), HP1γ (S93A) mutant, and HP1γ (S93E) mutant were ectopically expressed in 2BS fibroblasts, followed by RasV12-induced senescence. Without RasV12, expression of HP1γ mutants did not affect SAHF nor senescence levels (data not shown). After RasV12-induced senescence, HP1γ (wild-type) and HP1γ (S93E) mutant were distributed as large foci and colocalized with H3K9me3, a heterochromatic marker, whereas HP1γ (S93A) mutant was diffusely distributed throughout the nucleus in senescent cells (Fig. 4A). Consistent with this result, GST-tagged HP1γ (wild-type) and mutant wild-type HP1γ (S93E) effectively interacted with histone H3 and H3K9me3 in senescent cells, while mutant GST-HP1γ (S93A) did not (Fig. 4B). Furthermore, an *in vitro* experiment demonstrated that phosphorylation of HP1γ on Ser93 by rPIM-1 promoted it to bind to a biotinylated H3K9me2 peptide, whereas its S93A mutant did not have this effect (Fig. 4C). These data suggest that Ser93 phosphorylation enhances the capacity of HP1γ to bind to H3K9me3.

It has been shown that HP1γ associates with suppression of the CCNA2 and proliferating cell nuclear antigen (PCNA) promoters in senescent cells through a heterochromatin mechanism (Narita *et al*., 2003). We examined whether it is the phosphorylated form of HP1γ (pHP1γS93) that occupies these two genes’ promoters. A chromatin immunoprecipitation (ChIP) with anti-pHP1γS93 antibody revealed that HP1γ was enriched on the promoter sequence of CCNA2 and PCNA genes in senescent 2BS cells (Fig. 4D). Consistently, senescent cells with PIM-1 knockdown displayed a decreased pHP1γS93 levels on these promoters (Fig. 4D) and increased CCNA2 and PCNA expressions (Fig. 4E). In addition, phosphorylated form of HP1γ (pHP1γS93) that occupies these two genes’ promoters still occurred in BJ fibroblasts (Fig. S7). This suggests that pHP1γS93 is involved in gene silencing in senescent cells *in vivo*.

To further investigate whether HP1γ phosphorylation functionally is relative to cellular senescence, HP1γ mutants (S93A, S93E, or S93W) were ectopically expressed in 2BS fibroblasts, while shHP1γ was used to deplete endogenous HP1γ. HP1γ or mutant HP1γ (S93E) overexpression, without RasV12, did not induce SAHF or senescent phenotype (data not shown). RasV12-induced senescence was delayed by S93A mutant, but not S93E mutant or S93W HP1γ (Fig. 4F); this indicates that it is phosphorylated HP1γ that is associated with cells proliferation arrest.

### IL-6 signaling regulates PIM-1 expression during OIS

**Figure 5:**
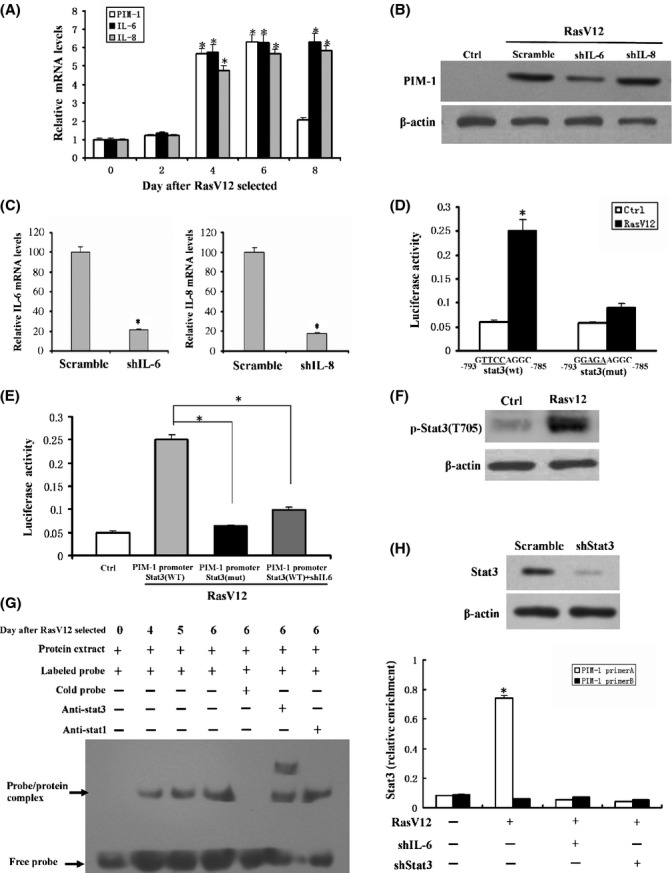
IL-6 signaling regulates PIM-1 expression during OIS. (A) Real-time PCR analysis of PIM-1, IL-6, and IL-8 expression in 2BS cells upon exposure to RasV12 at the indicated time. (B) Western blot analysis of PIM-1 expression in 2BS cells expressing IL-6 shRNA (shIL-6), IL-8 shRNA (shIL-8), or scramble upon exposure to RasV12. (C) Real-time PCR analysis of IL-6 and IL-8 expression in 2BS cells expressing IL-6 shRNA (shIL-6), IL-8 shRNA (shIL-8), or scramble at 6 days after RasV12 selected. (D) Luciferase assay examining the effect of STAT3 site mutations on the activity of PIM-1 promoter (the STAT3 binding site from −793 to −785 GTTCCAGGC was mutated to GGAGAAGGC) upon exposure to RasV12. (E) Luciferase assay examining the effect of IL-6 knockdown on the activity of the PIM-1 promoter in Ras-induced senescent cells. (F) Western blot analysis of the level of phosphorylation on Thr705 of STAT3 in Ras-induced senescent cells. (G) EMSA analysis of STAT3 DNA-binding capacity in 2BS cells upon exposure to RasV12. (H) ChIP analysis of STAT3 binding to PIM-1 promoter in Ras-induced senescent cells expressing scramble, shIL-6, or shSTAT3. ChIP enrichment was measured using real-time PCR, normalized by input DNA. Experiments from (B–F) and (H) were performed at 5 days after RasV12 selected. *, *P* < 0.05 (*t*-test).

Having identified PIM-1 as an important regulator of cellular senescence, we continued on to resolve the mechanism underlying the up-regulation of PIM-1 expression during senescence. Previous reports have shown that PIM-1 gene expression is induced by a large set of cytokines (Bachmann & Moroy, 2005), and many cytokine genes, including IL-6 and IL-8, are activated during senescence (Coppe *et al*., 2008). Thus, we reasoned that cytokine signaling is critically involved in the regulation of PIM-1 in senescent cells. Here, we chose to study IL-6 and IL-8, because these cytokines play a critical role in cellular senescence (Acosta *et al*., 2008; Kuilman *et al*., 2008). Dynamic analysis revealed that IL-6 and IL-8 expression coincided with the induction of PIM-1 expression (Fig. 5A). Subsequently, we determined whether depletion of each of these cytokines blocks the RasV12-induced PIM-1 expression. IL-6 knockdown with a validated shRNA evidently attenuated the RasV12 induction of PIM-1 expression, whereas shRNA against IL-8 had little, if any, effect on PIM-1 expression, suggesting that IL-6 is associated with PIM-1 expression in OIS (Fig. 5B). Figure 5(C) showed that IL-6 and IL-8 were effectively knocked down by their specific shRNA. The PIM-1 promoter contains a perfect consensus binding site from −793 to −758 bp for the transcription factor, STAT3, a downstream effecter of IL-6. To examine whether the IL-6/STAT3 pathway directly regulates PIM-1 expression at the transcriptional level, this binding site was mutated from GTTCCAGGC to GGAGAAGGC. Reporter analysis showed that RasV12 overexpression increased the wild-type PIM-1 promoter activity, and mutation of the STAT3 binding site dramatically attenuated the RasV12-induced promoter activity (Fig. 5D). In addition, expressing IL-6 shRNA suppresses the PIM-1 promoter activity (Fig. 5E). These data suggest that the IL-6/STAT3 pathway activates the transcription of PIM-1 gene. Subsequently, we determined whether STAT3 is activated as a function of oncogenic stress. Indeed, STAT3 phosphorylation at Tyr705, which is a marker of STAT3 activation, increased during OIS (Fig. 5F). An EMSA experiment also identified that the STAT3 (but not STAT1) DNA-binding capacity increased during OIS (Fig. 5G). ChIP assay was performed to confirm that STAT3 binds to the PIM-1 promoter in living cells. As expected, STAT3 bound to the PIM-1 promoter *in vivo* upon exposure of oncogenic stress (Fig. 5H). Depletion of STAT3 or IL-6 with shRNA strongly diminished this binding, further validating the specificity of this signaling pathway (Fig. 5H). Taken together, these data suggest that activation of IL-6/STAT3 signaling upregulates PIM-1 expression during OIS.

**Figure 6:**
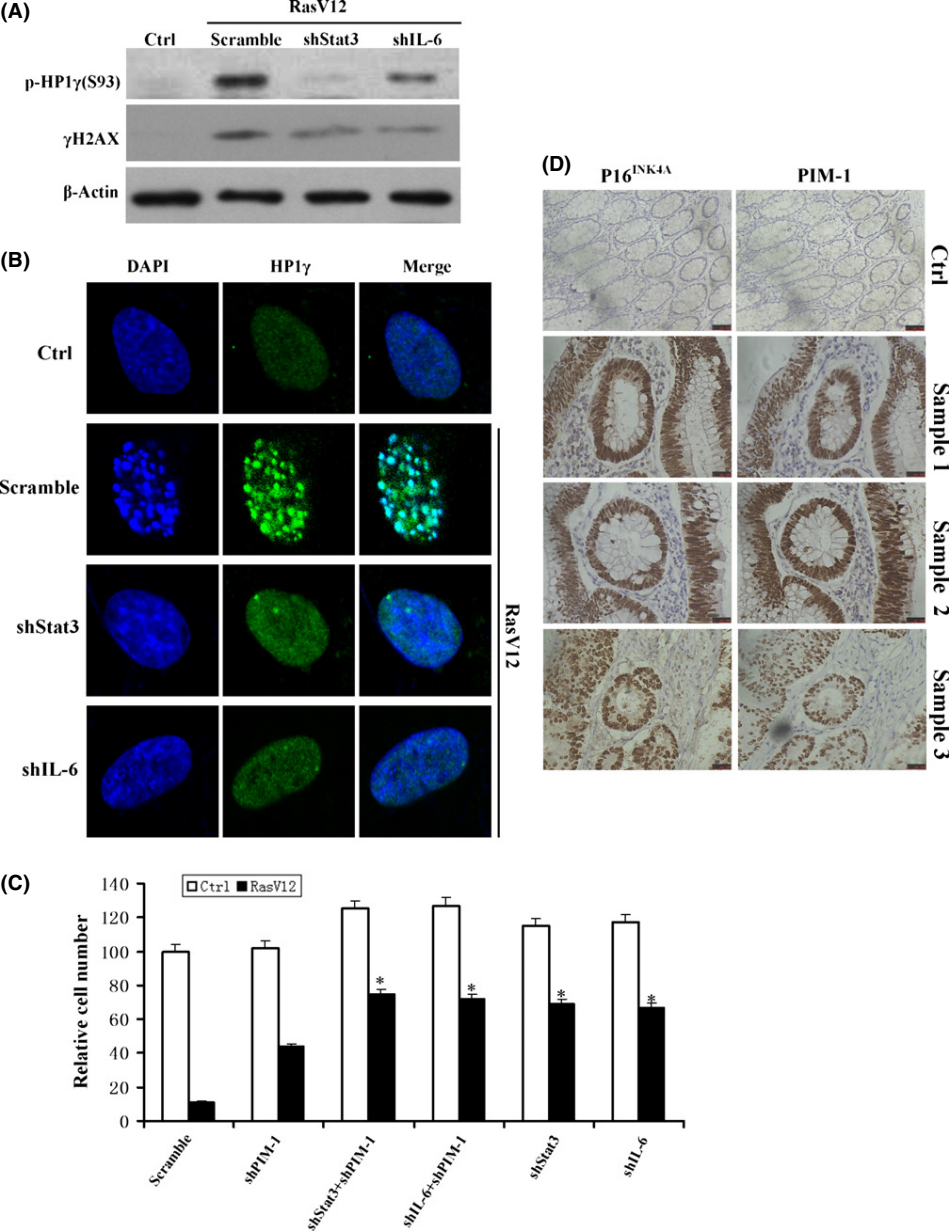
IL-6 signaling regulates HP1γ phosphorylation and SAHF formation during OIS. (A) Western blot analysis of the level of γH2AX and HP1γ phosphorylation at S93 (pHP1γS93) in 2BS cells expressing scramble, shSTAT3, or shIL-6 upon exposure to RasV12. (B) Confocal microscopy images of 2BS cells expressing scramble, shSTAT3, or shIL-6 upon exposure to RasV12. Immunofluorescence was performed for HP1γ (green), and DAPI staining (blue) was used to visualize DNA. (C) 2BS cells infected with the indicated vectors were seeded in 10-cm dishes. The plates were fixed 10 day after seeding and stained with crystal violet. Crystal violet was extracted and quantified and normalized by scramble control. *P < 0.05. (D) Three human colon adenomas and their adjacent normal tissues were immunostained for PIM-1 and the cellular senescence marker p16^INK^^4A^. Three representative colon adenomas and one normal control are shown.

Our data above raised the possibility that IL-6/STAT3 signaling has an essential role in DNA damage response and heterochromatin formation. Indeed, shRNA that successfully targeted IL-6 or STAT3 led to lower levels of γH2AX and pHP1γS93 expression (Fig. 6A) and decreased HP1γ foci formation upon oncogenic stress (Fig. 6B). It suggests that IL-6/STAT3 signaling functionally is associated with PIM-1 at least in OIS. To further explore whether PIM-1 and IL-6/STAT3 act epistatically, we analyzed the effects of shPIM-1/shIL-6, shPIM-1/shSTAT3, shPIM-1, shIL-6, or shSTAT3 on RasV12-induced senescence. Results showed that cells with shPIM-1/shIL-6 or shPIM-1/shSTAT3 co-expression displayed stronger proliferative capacity than cells with shPIM-1 alone, but no difference was observed between shPIM-1/shIL-6 or shPIM-1/shSTAT3 co-expression and shIL-6 or shSTAT3 alone (Fig. 6C), suggesting that besides of PIM-1, other factors also involved in IL-6 regulation of cellular senescence.

### PIM-1 expression is elevated in preneoplastic lesions

PIM-1 up-regulation during OIS prompted us to ask whether it might also be up-regulated in preneoplastic lesions associated with senescent cells. To this end, we monitored PIM-1 expression inhuman colon adenomas by immunohistochemical staining. We choose this lesion because it contains cell groups displaying two hallmarks of cellular senescence, strong p16^INK4A^ immunopositivity, and absence of both mitotic figures and Ki-67 (Dai *et al*., 2000). As shown in Fig. 6D, preneoplastic colon adenomas were enriched in senescent cells, which were judged by p16^INK4A^ staining. Furthermore, these samples stain positive for PIM-1, suggesting that PIM-1 contributes to OIS *in vivo*.

## Discussion

Proliferative arrest is a major characteristic of senescent cells. It is not difficult to understand why many anti-oncogenes are activated and exert a key role in cellular senescence. Increasing evidences have shown that cellular senescence acts as a protective mechanism against carcinogenesis (Lanigan *et al*., 2011; Hoenicke & Zender, 2012; Vargas *et al*., 2012). Unexpectedly, in this study, we found that the oncogene PIM-1 expression is induced during senescence and loss of PIM-1 can impair the cellular senescence response in normal fibroblasts, suggesting that PIM-1 probably has properties of a tumor suppressive role. Through searching the oncomine database (http://www.oncomine.org), we found that PIM-1 displays an under-expressed expression in some tumor tissue such as breast cancer, esophageal cancer and ovarian cancer (data not shown). Other studies have reported that a high PIM-1 level correlates with good prognosis in prostate adenocarcinoma, pancreatic ductal carcinoma, and nonsmall cell lung cancer (Dhanasekaran *et al*., 2001; Reiser-Erkan *et al*., 2008; Warnecke-Eberz *et al*., 2008). Another experiment supporting this view, although not relating to tumors, showed that PIM-1 plays a pro-death role in neurons exposed to DNA damage (Zhang *et al*., 2010), which is contrary to its pro-survival properties of dividing cells. In this sense, PIM-1 itself is part of the cellular senescence mechanism. Inhibition of PIM-1 expression alone probably induces tumorigenesis in normal cells upon activation of other oncogenic stress. Thus, one must be cautious when selecting PIM-1 inhibitor for cancer therapeutic intervention.

Our current study characterized that PIM-1 phosphorylates HP1γ on Ser93, and this modification promotes HP1γ to bind to H3K9m3 for heterochromatin formation in OIS. However, the same site, previous referred as Ser83 due to splicing variant, can also be phosphorylated by protein kinase A (PKA) in cancer cell lines (Lomberk *et al*., 2006). Lomberk *et al*. reported that p-Ser83-HP1γ has an exclusively euchromatic localization, impairs silencing activity, and serves as a marker for transcription elongation. These contradictory results raise the possibility that Ser93 phosphorylation may influence HP1γ localization and function through different combinations of other post-translational modifications. It has been shown that Thr51, located at the N-terminal chromodomain (CD) of HP1β, can be phosphorylated by CK2 and can reduce the affinity of HP1β for H3K9me3 binding (Ayoub *et al*., 2008). Given that CD is a highly conserved domain among the three HP1 isoforms and CK2 activity is decreased in senescent cells (Ryu *et al*., 2006), the absence of Thr51 phosphorylation may be conducive to pHP1γS93 binding to H3K9me3 and heterochromatin formation during senescence. In contrast, cancer cells have high CK2 activity (Hanif *et al*., 2010; Trembley *et al*., 2010). Hence, Thr51 phosphorylation may block pHP1γS93 binding to the chromatin via H3K9me3, shifting the HP1γ function from heterochromatin formation and gene silencing to euchromatic gene expression. Of course, other mechanisms regulating the localization of pHP1γS93 cannot be ruled out.

It has been shown that OIS is specifically linked to the activation of an inflammatory transcriptome, including pleiotropic cytokine IL-6 (Coppe *et al*., 2008). Besides its paracrine mitogenic action, IL-6 is required for the execution of OIS in a cell-autonomous mode (Kuilman *et al*., 2008). IL-6 depletion causes the inflammatory network to collapse and abolishes senescence entry and maintenance (Kuilman *et al*., 2008). Several pathways have been shown to contribute to the induction of IL-6 during OIS (Kuilman *et al*., 2008; Rodier *et al*., 2009; Chien *et al*., 2011); however, the mechanisms by which IL-6 regulates OIS have not been studied. In the current study, we demonstrated that IL-6 signaling might regulate DNA damage response and heterochromatin formation, which plays an important role in the induction and maintenance of cellular senescence respectively, at least partly through induction of PIM-1 expression. Interestingly, neither IL6 nor PIM1 knockdown can affect cell senescence or SAHF formation by itself without stress from RasV12. Kuilman *et al*. have also showed that IL-6 activated senescence by induction of p15-INK4b. Here, we observed an increased IL-6 level after overexpression of PIM-1 (Fig. 2E), which suggested an indirectly regulation between PIM-1 and p15 during cell senescence.

It is widely accepted that chromatin near a damaged site requires an ‘opened’ state for efficient recruitment of repair factors and subsequent repair (Price & D’Andrea, 2013). Thus, heterochromatin formation during senescence most likely prevents DNA repair factors from approaching damaged DNA sites, resulting in a persistent DNA damage response (DDR). It has been shown that DDR signaling triggers senescence-associated inflammatory cytokine secretion including IL-6 (Rodier *et al*., 2009). In prostate cancer cell lines, PIM1 was able to induce senescence, which is associated with DNA damage and activation of the p53 pathway (Zemskova *et al*., 2010). It is conceivable that IL-6 up-regulation promotes heterochromatin formation and thus leads to sustained DDR. This positive feedback loop between DNA damage response and IL-6, which is involved in heterochromatin, possibly plays a critical role in the maintenance of cellular senescence.

SAHF are thought to contribute to the irreversible cell cycle exit in many senescent cells by repressing the expression of proliferation-promoting gene such as *CCNA2*. Several proteins take part in the formation and/or maintenance of the SAHF, including histone chaperones HIRA and Asf1, HP1 and high-mobility group A (HMGA) proteins, histone variant macroH2A, and Rb. It has been shown that in cells entering senescence, HP1γ, but not the related proteins HP1α and HP1β, become phosphorylated on serine 93, and this phosphorylation is required for efficient incorporation of HP1γ into SAHF. We identified that kinase PIM-1 is responsible for this phosphorylation of HP1γ, and this modification is required for heterochromatin formation and silence of genes. However, it appears that this modification is not required for SAHF formation, because mutant HP1γS93E overexpression did not induce SAHF (data not shown), consisting with a previous report, which shows that reduction in the amount of HP1 proteins does not detectably affect chromosome condensation into SAHF. Thus, although PIM-1 knockdown suppresses SAHF formation, such effect likely is independent of HP1γ.

## Experimental procedures

### Cell culture and human samples

Human diploid fibroblasts 2BS and BJ cells were obtained from the National Institute of Biological Products, Beijing, China, and WI-38 cells were purchased from ATCC. Cells were cultured as described previously (de Zhuo *et al*., 2010). Paraffin-embedded grade-II tubulovillous colon adenoma (*n* = 3) and adjacent normal tissues (*n* = 3) specimens were collected at Peking University First Hospital.

### RNA interference

PIM-1, IL-6, IL-8, or STAT3 shRNAs were synthesized and cloned into pLVX-shRNA2 vector (Clontech, Mountain View, CA, USA). The resulting plasmids were used for generating lentiviruses. The sense strand of various shRNAs was available in Table S1. To produce shRNA lentivirus for target cells infection, the pLVX-shRNA plasmid was transfected into 293T packaging cells using the Lenti-X HTX Packaging System (Clontech). Virus-containing medium was collected, supplemented with 8 μg mL^−1^ of polybrene (Sigma, St. Louis, MO, USA), and incubated with target 2BS cells at 37 °C for 22 h. Infected cells were purified by drug selection (3 μg mL^−1^ puromycin).

### Cell proliferation assay

Cells were infected with shRNA-expressing lentivirus, briefly selected for successful proviral integration. 4 × 10^5^ cells with shRNA were seeded into 6-cm dish and subsequently infected with RasV12-encoding virus and selected. Cells were fixed and stained 10 days postselection with crystal violet.

### Luciferase assay

The PIM-1 promoter was cloned via PCR in pGL3-Basic reporter vector (Promega, Madison, WI, USA). The oligonucleotide sequences for PCR primers were listed in Table S1. The mutated PIM-1 promoter was constructed by the QuikChange site-directed mutagenesis kit (Stratagene, Santa Clara, CA, USA). For promoter activity assays, 2BS cells were seeded in a 24-well plate (1 × 10^5^ cells/well), incubated overnight in complete growth medium, and transfected with 0.45 μg of reporter plasmid and 0.05 μg of control reporter (pRL-CMV). After 24 h of incubation, cells were lysed in passive lysis buffer (Promega), and luciferase activity was measured using the luciferase assay system (Promega) with a luminometer (Lumat LB 9501; Berthold, Bad Wildbad, Germany). All luciferase assays were conducted at least three times in triplicate. Firefly luciferase activity was normalized to the Renilla luciferase activity for each transfected well.

### Quantitative RT-PCR

Quantitative RT-PCR (qRT-PCR) was performed on cDNA generated from total RNA using the protocol of the M-MLV Reverse Transcriptase kit (Invitrogen, Carlsbad, CA, USA). Amplification and detection of specific products were performed with the ABI Prism 7300 sequence detection system (Invitrogen) with the cycle profile according to the TOYOBO SYBR qRT-PCR Mix kit (Osaka, Japan). Primer sequences are indicated in Table S1.

### Immunoblotting

Western blot and immunoprecipitations were performed by standard method using the following antibodies: monoclonal mouse anti-human β-actin (Cell Signaling, Danvers, MA, USA; 4967), HP1γ (sc-365085) antibodies, polyclonal rabbit anti-human PIM-1 (Santa Cruz Biotechnology, Santa Cruz, CA, USA; sc-28777), Ras (Santa Cruz Biotechnology; sc-29), and p16 (Santa Cruz Biotechnology; sc-468) antibodies, polyclonal rabbit anti-human p53 (Cell Signaling; 9282), p21 (Cell Signaling; 2947), p-Rb (Cell Signaling; 9308), Rb (Cell Signaling; 9313), HP1γ(Cell Signaling; 2619), p-STAT3 (Cell Signaling; 9145), STAT3 (Cell Signaling; 4904), HMGA2 (Cell Signaling; 8179), pHP1γS93. Phosphor-specific HP1γ at Ser 93 (pHP1γS93) antibody was prepared as described previously (Lomberk *et al*., 2006).

### SA-β-gal, SAHF staining, and BrdU incorporation assay

SA-β-gal activity and SAHF staining were performed as described previously (de Zhuo *et al*., 2010). For BrdU labeling, coverslips were transferred into a 6-well plate and incubated in 2 mL of the culture medium containing 10 μm BrdU for 36 h. The cells were then fixed with 4% paraformaldehyde for 10 min. Cells were incubated with 2 MHCl at 37 °C for 20 min to denature the DNA. Neutralization of the DNA was conducted using Tris borate-EDTA buffer (pH 8.4). The cells were then blocked in Tris-buffered saline solution containing 1% bovine serum albumin and 0.1% triton X-100 for 1 h. Monoclonal antibody against BrdU (Sigma) was then added and incubated at 4 °C overnight. Cells were incubated with horseradish peroxidase-conjugated secondary antibody for 30 min. A solution containing 3,3′-diaminobenzidine tetraanhydrochloride (0.16 mg mL^−1^) and 0.3% H_2_O_2_ was then added and incubated for 30 min in the dark.

### Chromatin immunoprecipitation and EMSA

Chromatin immunoprecipitation (ChIP) was performed using a chromatin immunoprecipitation assay kit (Thermo Fisher Scientific, Waltham, MA, USA). Antibodies used for ChIP were anti-STAT3 (Cell Signaling) and antiphospho-HP1γ. Primer pairs used for PCR were described in Table S1. Electrophoretic mobility shift assays (EMSA) were performed using the Light Shift Chemiluminescent EMSA Kit (Thermo) and Biotin 3′ End DNA Labeling Kit (Thermo). The oligonucleotide sequence of the DNA probe was described in Table S1.

### GST-HP1γ fusion protein purification and *in vitro* phosphorylation assays

GST and GST-HP1γ fusion protein expression was induced in BL21 cells (Stratagene) by the addition of 2 mm isopropyl-b-D-thiogalactopyranoside and incubation for 2 hr at 37 °C. Cells were lysed and subsequently purified using glutathione Sepharose 4B affinity chromatography in accordance with the manufacture instructions. For *in vitro* phosphorylation assays, fusion proteins were incubated with recombinant kinase rPIM-1 (Abnova, Taipei, Taiwan) for 20 min at 22 °C in buffer containing 25 mm HEPES-KOH, pH 7.5, 30 mm NaCl, 10 mm MgCl_2_, 0.5 mm dithiothreitol, and 10 μCi (3000 Ci mmol^−1^) of [γ-^32^P]ATP. The reaction mixture was boiled in Laemmli buffer, and phosphorylated proteins were separated by 12% SDS-PAGE followed by autoradiography.

### H3K9me2 binding assays

H3K9me2-binding assays were carried out with biotinylated H3K9me2 peptide (Upstate Biotechnologies, Lake Placid, NY, USA; 12-404) attached to streptavidin agarose beads (Pierce, Rockford, IL, USA; 20347). Binding assays were carried out with 4 nmol H3K9me2 peptide and 2 μg bacterially expressed recombinant GST-HP1γ protein (or its S93A mutant form), and 20 μL of 50% streptavidin agarose beads was added to a PBS-based-binding buffer containing 0.2% NP-40 and 0.1% BSA and incubated at room temperature for 1 h. For phosphorylation assay, a 25 μL kinase reaction contains 2 μg GST–HP1 proteins, and 1 μg rPIM-1 in PIM-1 buffer with 10 μM ATP. After binding, the mixture was centrifuged at 5000 *g* for 10 min, and all supernatant removed by gentle aspiration. GST-HP1γ pull-down by the agarose beads was detected by western blot with HP1γ antibody and was used to judge the binding of HP1γ to H3K9me2.

### Immunofluorescence

Cells fixed with 4% formaldehyde were incubated with phosphate-buffered saline containing 0.1% triton X-100 and 2% bovine serum albumin for 1 h at room temperature, followed by incubation with antibodies specific for HP1γ (1:500; Santa Cruz) and H3K9me3 (1:500; CST) overnight at 4 °C. Cells were then washed with phosphate-buffered saline plus 0.1% triton X-100 and incubated with a fluorophore-conjugated secondary antibody. Cells were imaged on a Zeiss LSM 410 confocal laser-scanning microscope with ×60 magnification.

## Supporting Information

**Method S1** Plasmid and retroviruses. **Table S1** Oligonucleotides used in this study. **Fig. S1** PIM-1 Expression is Up-regulated in senescent WI38 cells. **Fig. S2** Ectopic Expression of PIM-1 Causes Premature Senescence. **Fig. S3** PIM-1 depletion delays cellular senescence in BJ cells. **Fig. S4** *PIM-1* knockdown diminishes the DNA-Damage Response of cells to RasV12. **Fig. S5** Anti-pHP1γS93 antibody reacts in western blot analysis with the WT HP1γ protein but not with the S93A mutant, when exposed to rPIM-1 (top panel). **Fig. S6** PKA activity in young and Ras-induced senescent cells. **Fig. S7** Phosphorylated HP1γ that is associated with cellular senescence.
