# Supplementary material for: PIM-1 modulates cellular senescence and links IL-6 signaling to heterochromatin formation
Source: Aging Cell. 2014 Jul 18;13(5):879–89. doi: 10.1111/acel.12249 (PMC4331745; doi:10.1111/acel.12249)
Supplement: Supplementary file 1 — Method S1 Plasmid and retroviruses. Table S1 Oligonucleotides used in this study. Fig. S1 PIM-1 Expression is Up-regulated in senescent WI38 cells. Fig. S2 Ectopic Expression of PIM-1 Causes Premature Senescence. Fig. S3 PIM-1 depletion delays cellular senescence in BJ cells. Fig. S4 PIM-1 knockdown diminishes the DNA-Damage Response of cells to RasV12. Fig. S5 Anti-pHP1γS93 antibody reacts in western blot analysis with the WT HP1γ protein but not with the S93A mutant, when exposed to rPIM-1 (top panel). Fig. S6 PKA activity in young and Ras-induced senescent cells. Fig. S7 Phosphorylated HP1γ that is associated with cellular senescence. [file acel0013-0879-sd1.docx]

**Revised ACE-13-0414 R2**

**PIM-1 modulates cellular senescence and links IL-6 signaling to heterochromatin formation**

**Supplementary Experiment Procedure**

**Plasmid and retroviruses**

The following plasmids were used for generating retroviruses: pBABE- neo H-RasV12 (gift of Xiaowei Zhang); pBABE-EGFP-HP1γ, pBABE-GST-HP1γ and pBABE-PIM-1 were prepared as follows: HP1γ cDNA was generated by PCR and cloned into the EGFP-C1 vector (Clontech, Mountain View, CA, USA) or pFN2A (GST) vector (Promega, Madison. WI, USA), and then EGFP-HP1γ or GST-HP1γ was subcloned into pBABE-puro vector (Addgene, Cambridge, MA, USA). The primers for PCR were listed in Table S1. PIM-1 cDNA insert from pDONR223-PIM-1 plasmid (Addgene) was sub-cloned in pBABE-puro vector. Mutants of pBABE-EGFP-HP1γ (S93E or S93A), pBABE-GST-HP1γ (S93E or S93A) and pBABE-K67M were constructed using the QuikChange site-directed mutagenesis kit (Stratagene,La Jolla, CA, USA).

Retroviral-mediated gene transfer was performed using the Phoenix packaging cells. Briefly, Phoenix cells were transfected by the Lipofectamine^TM^ 2000 method with retroviral plasmid DNA and a plasmid encoding vesicular stomatitis virus glycoprotein (VSV-G). Virus-containing medium was collected, supplemented with 8 μg/ml polybrene, and incubated with 2BS cells at 37^0^C for 24 hr. Frequently, a second round of infection was performed on the same target cells. Infected cells were purified by drug selection (3μg/ml pyromycin or 500μg/ml G418)

**Supplementary Table**

**
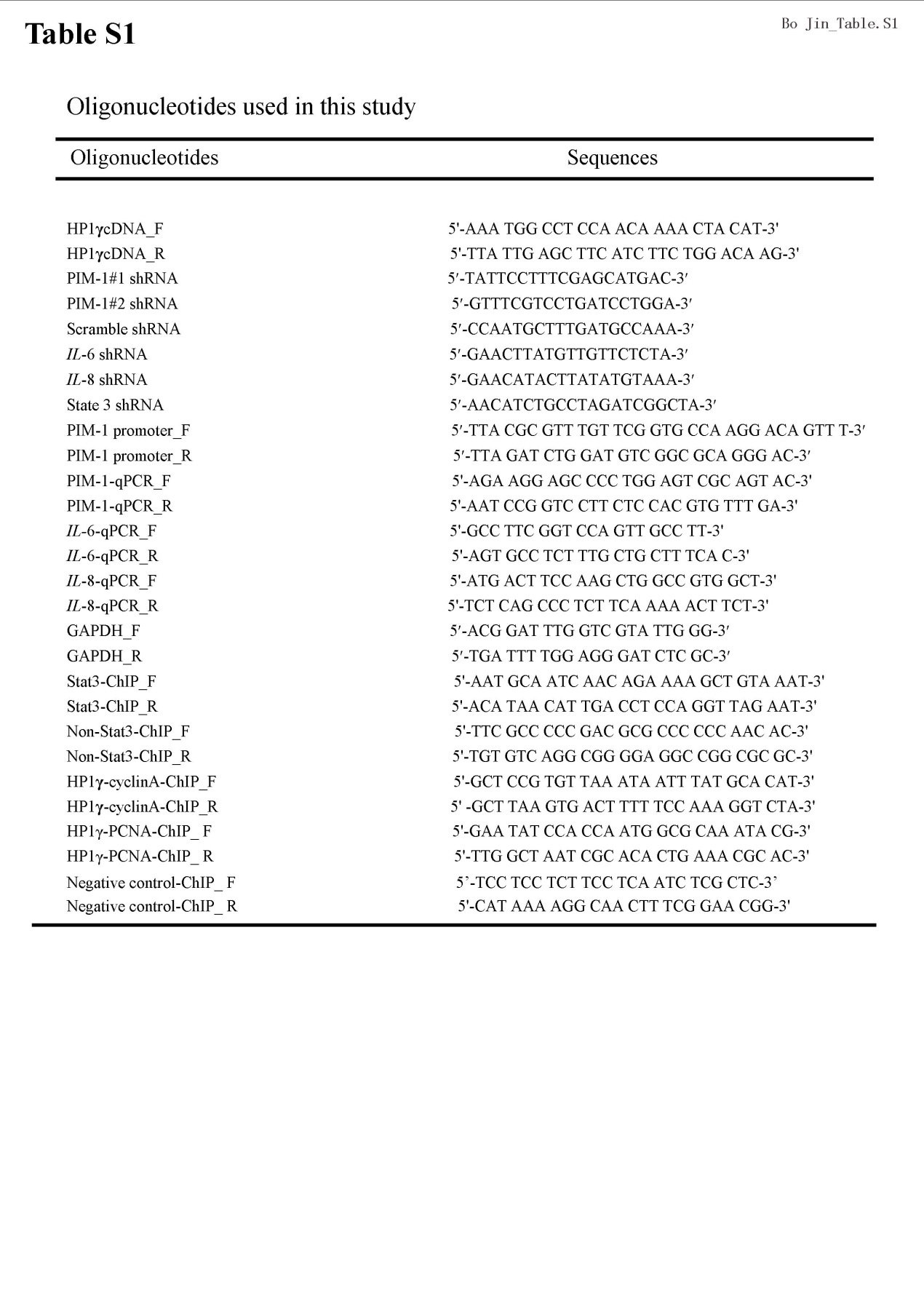
**

**Supplementary figure**

**
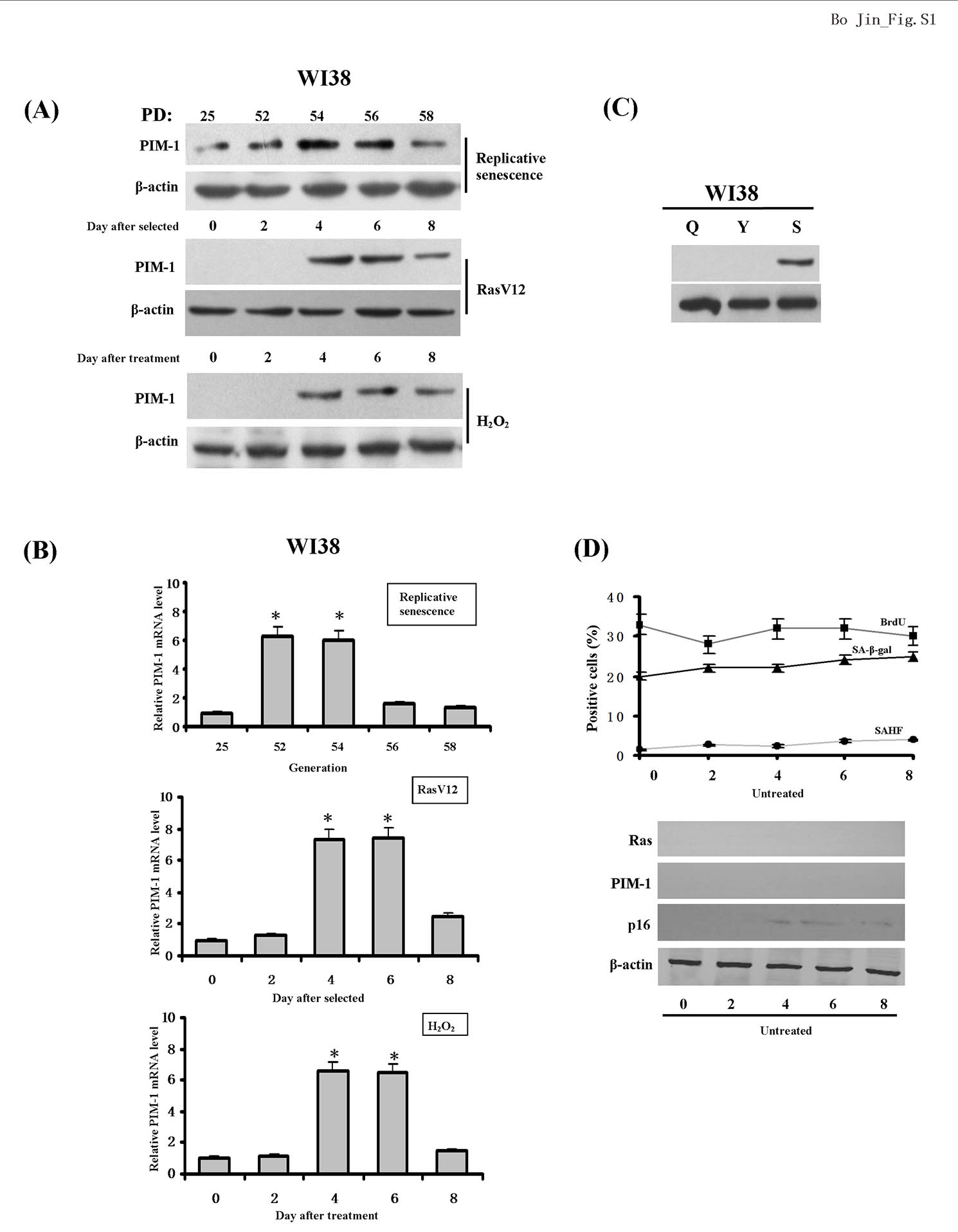
**

**Figure S1.** PIM-1 Expression is Up-regulated in Senescent WI38 Cells.Western blot (A) and real-time PCR analysis (B) of PIM-1 expression in replicative senescent, Ras-induced, andH_2_O_2_-induced premature senescent WI-38 cells. (C) Western blot analysis of PIM-1 expression in WI38 cells that are young (Y), quiescent (Q) by serum-deprivation, and senescent (S) by Ras induction. ß-actin was used as loading control. Data are expressed as the mean ± SD of three independent experiments. *, p< 0.05 (t test). (D) Young 2BS cells were assessed for SA-β-gal, BrdU incorporation, SAHF formation, and expression of the indicated proteins at the various time points without any treatment. Each value of SA-β-gal, BrdU incorporation and SAHF formation represents the mean + SD. (D) Young 2BS cells were assessed for SA-β-gal, BrdU incorporation, SAHF formation, and expression of the indicated proteins at the various time points (days) without any treatment. Each value of SA-β-gal, BrdU incorporation and SAHF formation represents the mean + SD.

**
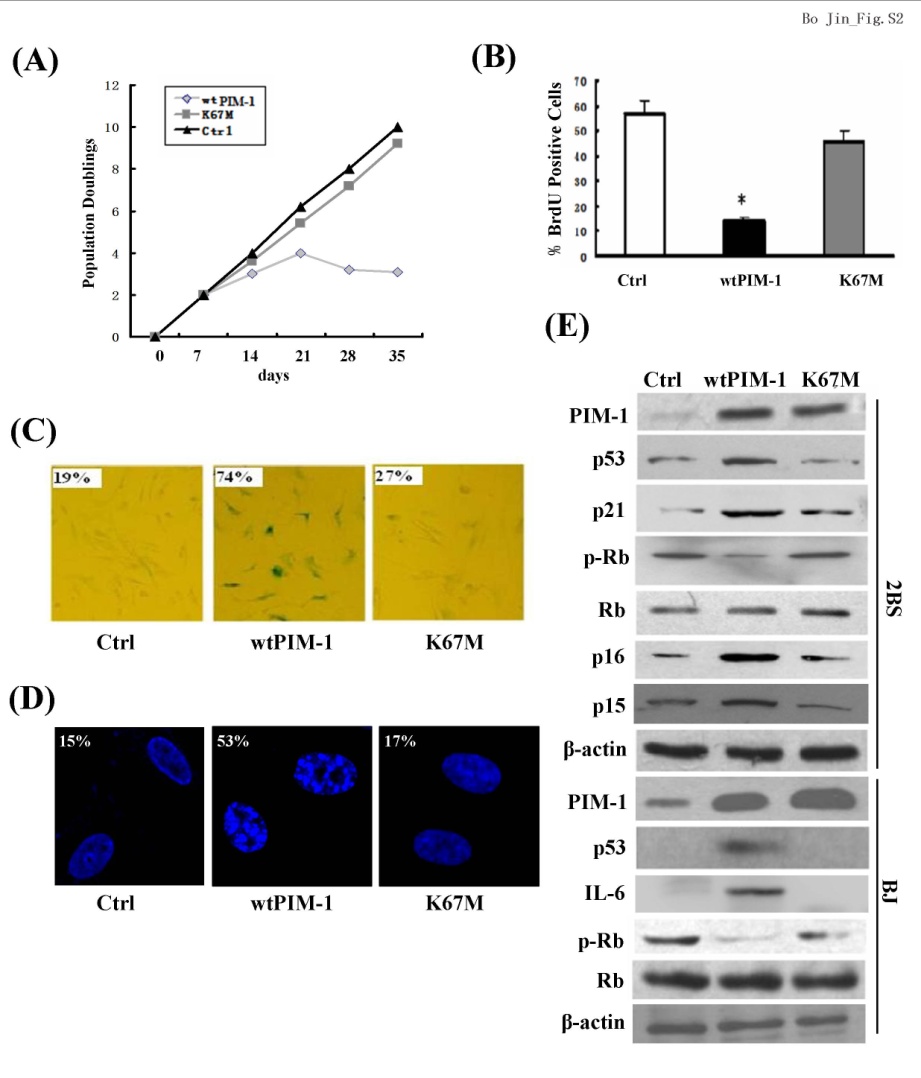
**

**Figure S2.**Ectopic Expression of PIM-1 Causes Premature Senescence.(A) 2BS cells were infected at passage 20 with control vectors, wild type PIM-1 (wtPIM-1) or mutant PIM-1 (K67M), selected, and growth curves were performed. (B) Percentage of BrdU-positive cells in 2BS cells infected with the indicated vector. Data are represented as mean + SD. *, p < 0.05 (t test). (C) SA-β-gal staining of 2BS fibroblast infected with the indicated vector. Percentages of SA-β-gal -positive cells are indicated. (D) DAPI staining of 2BS fibroblast infected with the indicated vector. Percentages of SAHF-positive cells are indicated. (E) 2BS and BJ cells expressing the control vector, or wtPIM-1 or K67M were analyzed for expression of the indicated proteins by western blot.

**
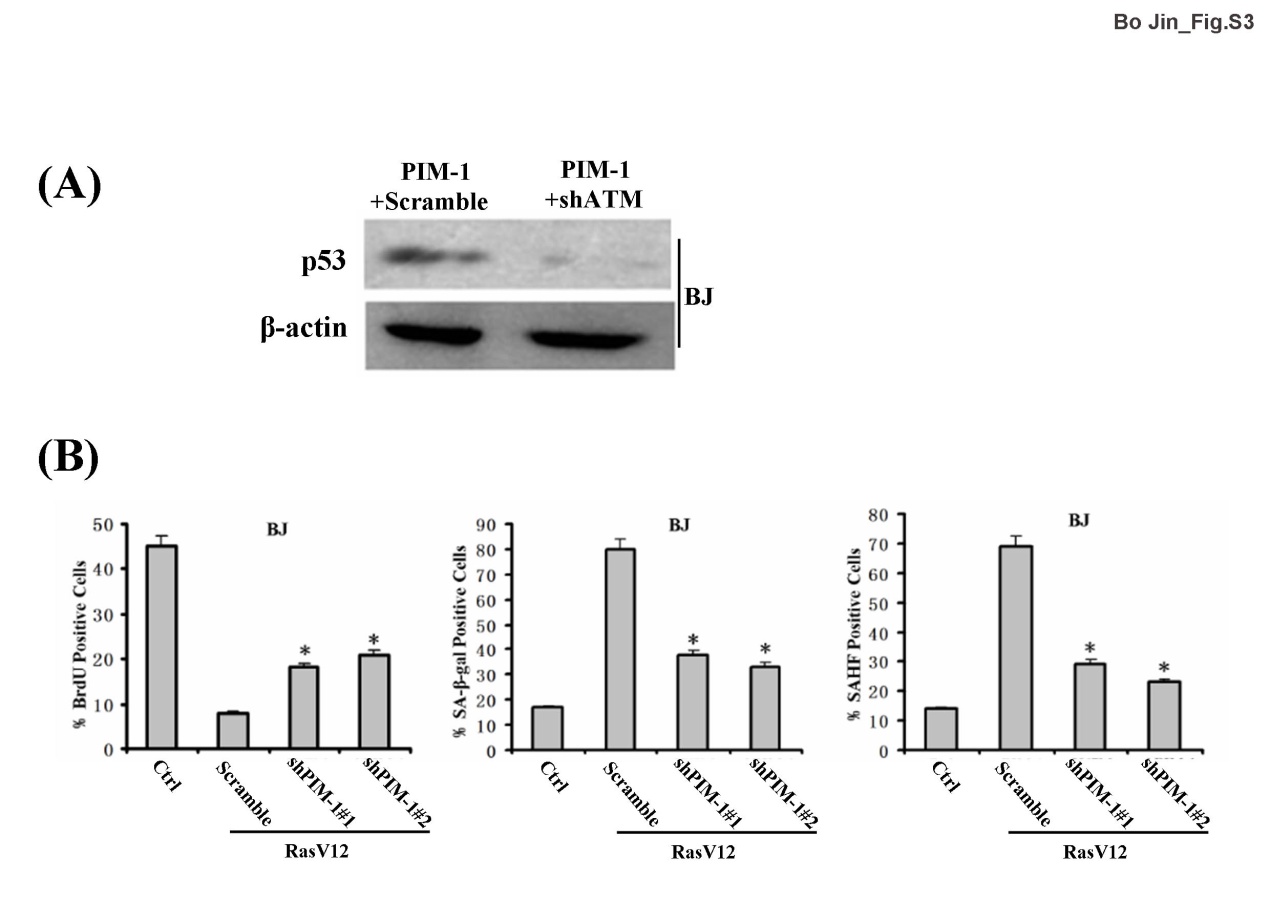
Figure S3.**PIM-1 depletion delays cellular senescence in BJ cells. (A) BJ cells expressing the PIM-1 and Scramble, or PIM-1 and shATMwere analyzed for expression of P53 proteins by western blot. (B) BrdU incorporation, SA-β-gal activity and SAHF formation of BJ cells expressing PIM-1 shRNAs upon exposure to RasV12. Data are represented as mean + SD. *, p < 0.05 (t test).

**
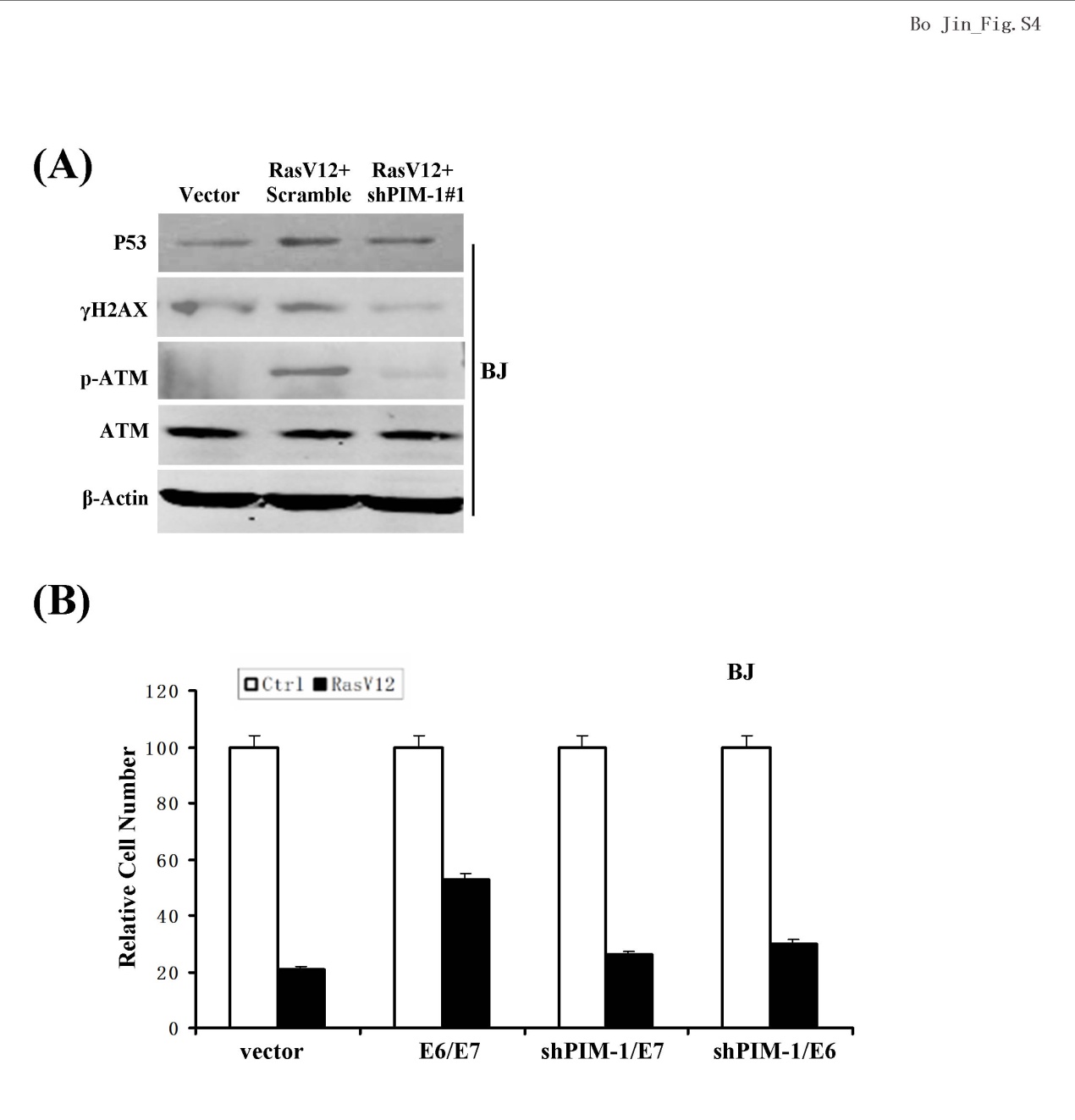
**

**Figure S4. *PIM-1* knockdown diminishes the DNA-Damage Response of cells to RasV12.** (A) Western blot of the indicated proteins expression inBJ cells expressing PIM-1 shRNA#1 at 12 days after exposure to RasV12. (B) BJ cells infected with the indicated vectors were seeded in 10cm dishes. The plates were fixed 10d after seeding and stained withcrystal violet. Crystal violet was extracted and quantified.


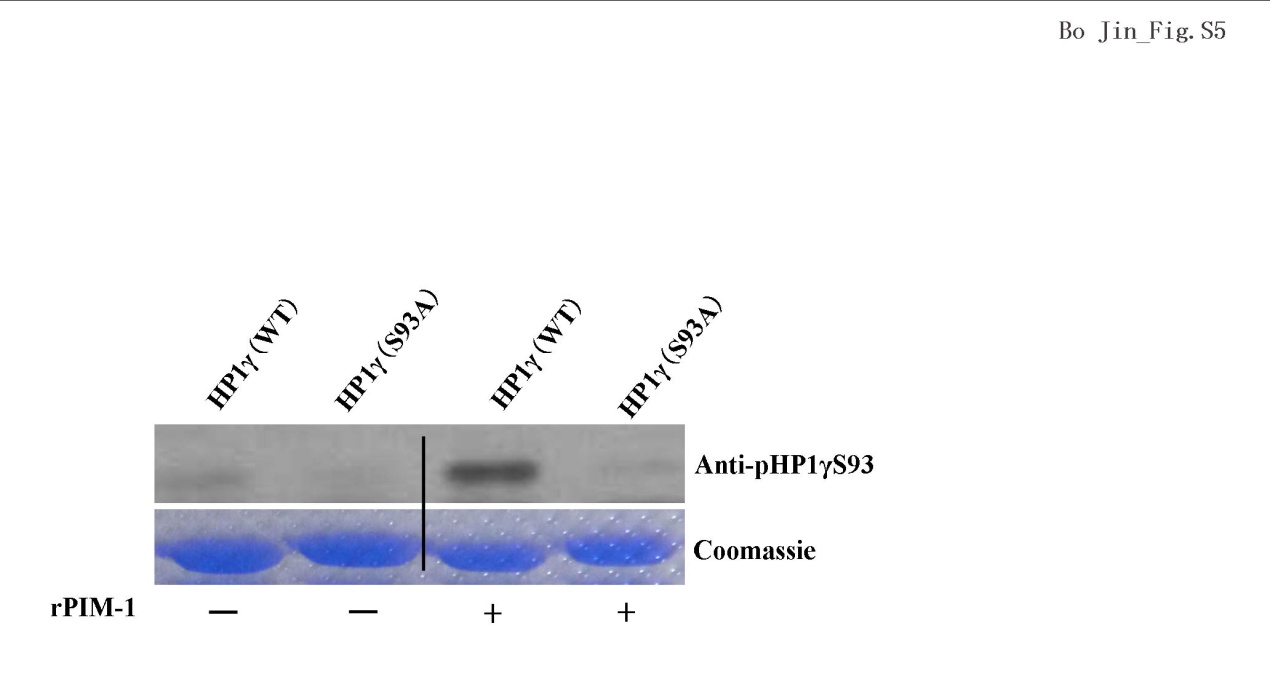


**Figure S5**.Anti-pHP1γS93 antibody reacts in western blot analysis with the WT HP1γ protein but not with the S93A mutant, when exposed to rPIM-1 (top panel). Coomassie is shown as a loading control (bottom panel).


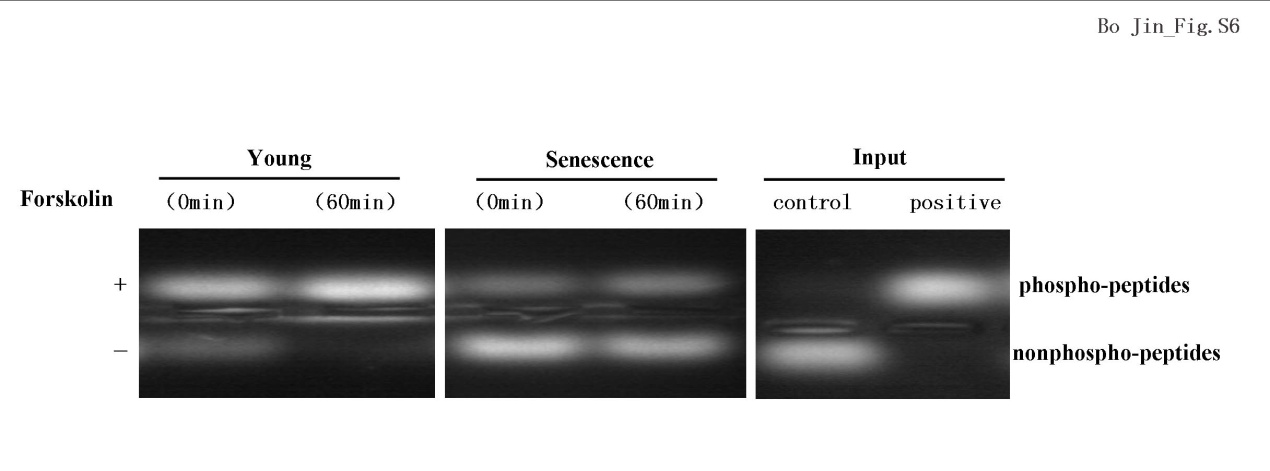


**Figure S6.** PKA activity in young and Ras-induced senescent cells. Cells were treated with Forskolin (20 μM) for 0 and 60 min. Crude extracts were prepared from young and senescent cells at 0 and 60 min after Forskolin (20 μM) treatment, and PKA activity was detected by the PepTag Assay for non-radioactive detection of cAMP-dependent protein kinase, a kit purchased from Promega Corporation. Phosphorylation of PepTag A1 peptide (PKA-specific peptide substrate) is used to judge PKA activity, where phosphorylated peptides migrates toward the positive electrode, while the non-phosphorylated substrate migrates toward the negative electrode.


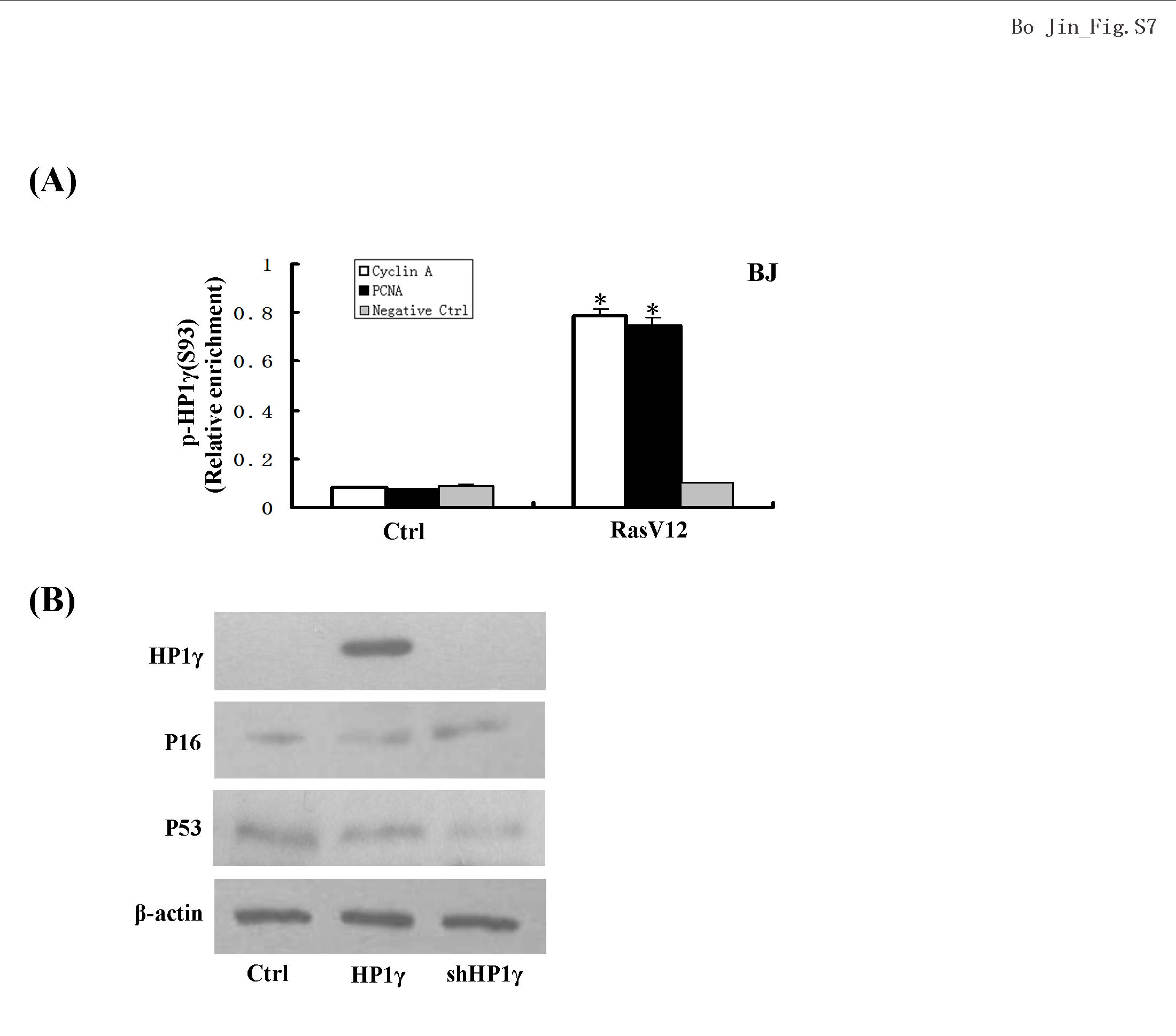


**Figure S7.** Phosphorylated HP1γ that is associated with cellular senescence. (A) ChIP analysis of phosphorylated HP1γ (pHP1γS93) binding to cyclin A and PCNA promoter in 2BS cells that expressing vector (Ctrl) or RasV12 (RasV12) in BJ fibroblasts. ChIP enrichment was measured using real-time PCR, normalized by input DNA. (B) 2BS cells expressing the control vector, HP1γ, or shHP1γ were analyzed for expression of the HP1γ, P16 or P53 levels by western blot.
